# A case of simultaneous diagnosis of tunica vaginalis testis and pleural mesothelioma

**DOI:** 10.1002/rcr2.937

**Published:** 2022-03-22

**Authors:** Rei Matsuki, Satoru Ishii, Tomoyuki Suzuki, Haruhito Sugiyama

**Affiliations:** ^1^ Department of Respiratory Medicine National Center for Global Health and Medicine Tokyo Japan

**Keywords:** FDG‐PET, pleural mesothelioma, thoracoscopy under local anaesthesia, tunica vaginalis testis mesothelioma

## Abstract

A 37‐year‐old man was admitted to our hospital with chest pain and fever. Computed tomography showed pleural effusion and irregularly marginated, elevated lesions inside diffuse pleural thickening. Detailed medical examination showed swelling of the left testicle. 18F‐fluorodeoxyglucose positron emission tomography showed uptakes at the thickened pleura and left testis. Pelvic magnetic resonance imaging showed a mass in the left testis with a heterogeneous and partially calcified tumour present interiorly. Thoracoscopy was performed under local anaesthesia, enabling the observation of masses at the pleura and biopsy of the mass, which was diagnosed as malignant pleural mesothelioma. The affected testicle was resected and diagnosed as tunica vaginalis testis mesothelioma. Thus, simultaneous tunica vaginalis testis and pleural mesothelioma were diagnosed. It is necessary to closely examine parts of the body other than the chest.

## Graphical abstract

Malignant pleural mesothelioma is one of the most severe diseases with a poor prognosis. Pulmonologists often make diagnoses of pleural mesothelioma with a history of asbestos exposure. It mainly involves the pleura, but can arise in other places, such as the tunica vaginalis testis. Thus, a case of simultaneous diagnosis of tunica vaginalis testis mesothelioma and malignant pleural mesothelioma is reported.

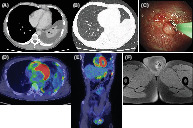

## INTRODUCTION

Malignant pleural mesothelioma is one of the most severe diseases with a poor prognosis. Diagnostic yields for malignant pleural mesothelioma of 38.7% from a combination of pleural effusion cytology and blind pleural biopsy and of 98% from thoracoscopy under local anaesthesia have been reported.^1^ Pulmonologists often make diagnoses of pleural mesothelioma with a history of asbestos exposure. It mainly involves the pleura, but can arise in other places, such as the tunica vaginalis testis. Thus, a case of simultaneous diagnosis of tunica vaginalis testis mesothelioma and malignant pleural mesothelioma is reported.

## CASE REPORT

**FIGURE 1:**
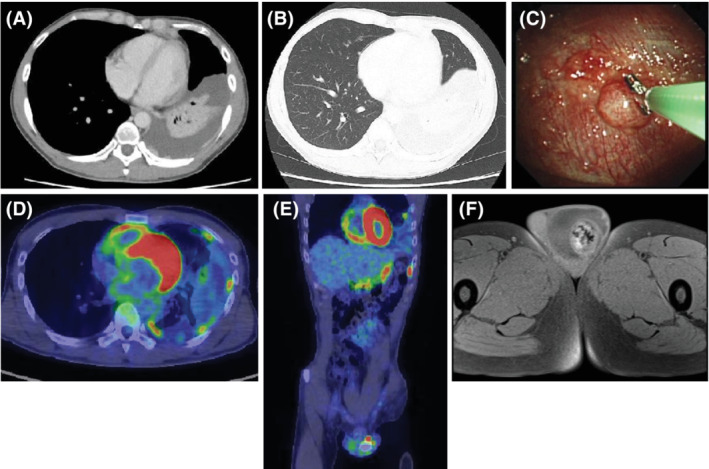
Computed tomography (CT) shows pleural effusion in the left lung and irregularly marginated, elevated lesions inside diffuse pleural thickening in the mediastinum (A). CT also shows an infiltrative shadow due to left lower lobe pressure‐drained atelectasis caused by pleural effusion in the lung field (B). To enable visualization of the masses at the pleura, biopsy forceps are inserted and moved near the mass (C). 18F‐fluorodeoxyglucose positron emission tomography‐CT shows uptakes at the thickened pleura and the left testis (D, E). Pelvic magnetic resonance imaging shows a 43 × 35 mm^2^ mass in the left testis with a heterogeneous, partially calcified tumour present interiorly. It also shows fluid accumulation in the scrotum (F)

**FIGURE 2:**
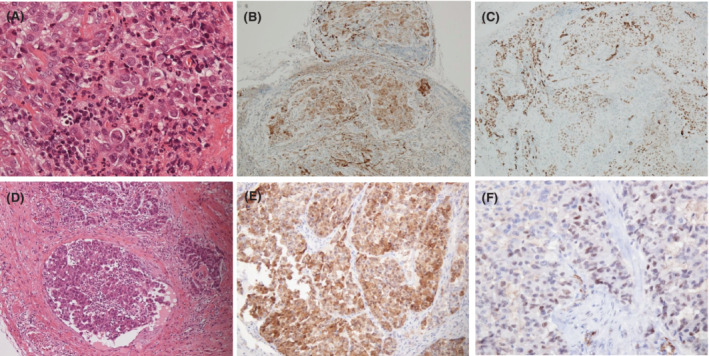
Histological analysis of the pleural tumour (A–C) and testicular tumour (D–F). Haematoxylin–eosin (HE) staining shows that atypical cells with distinct nucleoli and anisocytosis of the nucleus are proliferating and mixed with inflammatory cells (A). HE staining shows that tumour cells similar to pleural tissue are found. The tumour has spread to cover the inner surface of the capsule and is continuous with the tunica vaginalis testis (D). On immunohistochemical examination of the tissue, it is positive for calretinin (B, E) and for Wilms' tumour‐1 (C, F)

A 37‐year‐old man with no history of asbestos exposure presented with left chest pain and fever. Chest computed tomography showed pleural effusion and pleural thickening of the left lung (Figure 1A,B). Detailed medical examination showed that the left testicle was swollen. Therefore, 18F‐fluorodeoxyglucose positron emission tomography (FDG‐PET) and pelvic magnetic resonance imaging (MRI) examination were performed. FDG‐PET showed uptakes at the thickened pleura and the left testis (Figure 1D,E). Pelvic MRI showed a 43 × 35 mm^2^ mass in the left testis with a heterogeneous, partially calcified tumour present interiorly. It also showed fluid accumulation in the scrotum (Figure 1F). Thoracentesis showed that the effusion was exudative, and pleural cytodiagnosis was negative. Malignant pleural disease was suspected, and thoracoscopy under local anaesthesia was performed using the LTF‐240 (Olympus, Tokyo, Japan). Thoracoscopy showed the masses at the pleura, and biopsy followed. Haematoxylin–eosin (HE) staining showed that atypical cells with distinct nucleoli and anisocytosis of the nucleus were proliferating and mixed with inflammatory cells (Figure 2A).

On immunohistochemical examination of the tissue, it was positive for calretinin (Figure 2B) and Wilms' tumour‐1 (WT‐1) (Figure 2C). Moreover, it was positive for D2‐40 and CAM5.2, and negative for EMA, desmin, Napsin A, Ber‐EP4 and TTF‐1. The diagnosis was malignant pleural mesothelioma.

Malignant disease was also suspected in the testicle, and the left testicle was resected. HE staining showed tumour cells similar to pleural tissue. The tumour had spread to cover the inner surface of the capsule and was continuous with the tunica vaginalis testis (Figure 2D). On immunohistochemical examination of the tissue, it was positive for calretinin (Figure 2E) and WT‐1 (Figure 2F), as well as CAM5.2, but negative for Ber‐EP4.

## DISCUSSION

Simultaneous tunica vaginalis testis and malignant pleural mesothelioma were diagnosed. To the best of our knowledge, this is the first such report. A review article of 4710 mesothelioma cases in Europe and the United States reported that the sites of mesothelioma were pleura in 4181 (88.8%), peritoneum in 454 (9.6%), pleuroperitoneum in 30 (0.6%), pericardium in 33 (0.7%), testicular in nine (0.2%) and unknown in three (0.1%).^2^ The tunica vaginalis testis is a two‐layered serosal membrane that covers the anterior surface of the testis and epididymis. Only 223 cases of primary testicular mesothelioma had been reported worldwide by 2010.^3^ A review article of 73 cases reported that most of the patients were 55–75 years old, but 10% of the patients were younger than 25 years old. It has also been reported that 34.2% of the patients had a history of asbestos exposure, and past asbestos exposure was a risk factor as for malignant pleural mesothelioma.^4^ The present patient was young and had no asbestos exposure, but he may have had other minor risk factors like simian virus 40, although whether he was infected by it was not known.

In the present case, the histology of the two mesotheliomas was the same. Which one was the primary was not evident. Anatomically, it is presumed that tumour metastasized from the tunica vaginalis testis to the lymphatic trunk, thoracic duct, lymph nodes in the left chest and the left pleura. On the other hand, Ascoli et al. reported metastasis from pleura to the tunica vaginalis testis 1 year after the onset of a pleural effusion.^5^ It was difficult to distinguish which was the primary cancer or if it was a case of double cancers.

This time, it was found that there was not only pleural mesothelioma, but also tunica vaginalis testis mesothelioma based on a detailed medical examination and image inspection.

Some hospitals cannot perform imaging such as FDG‐PET. We should keep in mind that mesothelioma does not arise only from the pleura, but also from other organs such as the tunica vaginalis testis. However, patients who have symptoms such as swelling in the testis do not tell doctors about the symptoms because of embarrassment. It is possible that, in the present case, one could have only focused on the chest without a detailed medical examination.

In conclusion, a case of simultaneous diagnosis of testicular mesothelioma and pleural mesothelioma was reported. It is necessary for us to closely examine parts of the body other than the chest in such cases.

## CONFLICT OF INTEREST

None declared.

## ETHICS STATEMENT

The authors declared that appropriate written informed consent was obtained for publication of this manuscript and accompanying images.

## Data Availability

Data sharing is not applicable to this article as no data sets were generated or analysed during the current study.

## References

[1] Lee P , Hsu A , Lo C , Colt HG . Prospective evaluation of flexrigid pleuroscopy for indeterminate pleural effusion: accuracy, safety, and outcome. Respirology. 2007;12:881–6. 17986118 10.1111/j.1440-1843.2007.01144.x

[2] Gunnar H . Malignant mesothelioma 1982: review of 4710 published cases. Br J Dis Chest. 1983;77:321–43. 6357260

[3] Bisceglia M , Dor DB , Carosi I , Vairo M , Pasquinelli G . Paratesticular mesothelioma. Report of a case with comprehensive review of the literature. Adv Anat Pathol. 2010;17:53–70. 20032640 10.1097/PAP.0b013e3181c66fbc

[4] Plas E , Riedl CR , Pflüger H . Malignant mesothelioma of the tunica vaginalis testis. Cancer. 1998;83:2437–46. 9874447 10.1002/(sici)1097-0142(19981215)83:12<2437::aid-cncr6>3.0.co;2-g

[5] Ascoli V , Facciolo F , Rahimi S , Scalzo CC , Nardi F . Concomitant malignant mesothelioma of the pleura, peritoneum, and tunica vaginalis testis. Diagn Cytopathol. 1996;14:243–8. 8732655 10.1002/(SICI)1097-0339(199604)14:3<243::AID-DC9>3.0.CO;2-I

